# Impact of Water Quality, Sanitation, Handwashing, and Nutritional Interventions on Enteric Infections in Rural Zimbabwe: The Sanitation Hygiene Infant Nutrition Efficacy (SHINE) Trial

**DOI:** 10.1093/infdis/jiz179

**Published:** 2019-04-20

**Authors:** Elizabeth T Rogawski McQuade, James A Platts-Mills, Jean Gratz, Jixian Zhang, Lawrence H Moulton, Kuda Mutasa, Florence D Majo, Naume Tavengwa, Robert Ntozini, Andrew J Prendergast, Jean H Humphrey, Jie Liu, Eric R Houpt

**Affiliations:** 1 Department of Public Health Sciences, University of Virginia, Charlottesville; 2 Division of Infectious Diseases and International Health, University of Virginia, Charlottesville; 3 Zvitambo Institute for Maternal and Child Health Research, Harare, Zimbabwe; 4 Department of International Health, Johns Hopkins Bloomberg School of Public Health, Baltimore, Maryland; 5 Blizard Institute, Queen Mary University of London, United Kingdom

**Keywords:** enteric infections, sanitation, hygiene, stunting, environmental enteric dysfunction

## Abstract

**Background:**

We assessed the impact of water, sanitation, and hygiene (WASH) and infant and young child feeding (IYCF) interventions on enteric infections in the Sanitation Hygiene Infant Nutrition Efficacy (SHINE) trial in rural Zimbabwe.

**Methods:**

We tested stool samples collected at 1, 3, 6, and 12 months of age and during diarrhea using quantitative molecular diagnostics for 29 pathogens. We estimated the effects of the WASH, IYCF, and combined WASH + IYCF interventions on individual enteropathogen prevalence and quantity, total numbers of pathogens detected, and incidence of pathogen-attributable diarrhea.

**Results:**

WASH interventions decreased the number of parasites detected (difference in number compared to non-WASH arms, –0.07 [95% confidence interval, –.14 to –.02]), but had no statistically significant effects on bacteria, viruses, or the prevalence and quantity of individual enteropathogens after accounting for multiple comparisons. IYCF interventions had no significant effects on individual or total enteropathogens. Neither intervention had significant effects on pathogen-attributable diarrhea.

**Conclusions:**

The WASH interventions implemented in SHINE (improved pit latrine, hand-washing stations, liquid soap, point-of-use water chlorination, and clean play space) did not prevent enteric infections. Transformative WASH interventions are needed that are more efficacious in interrupting fecal–oral microbial transmission in children living in highly contaminated environments.

## Teaser

The water, sanitation, and hygiene and infant and young child feeding interventions tested in the Sanitation Hygiene Infant Nutrition Efficacy (SHINE) trial in rural Zimbabwe had no effects on enteric infections or pathogen-specific causes of diarrhea.

**(See the Editorial Commentary by Black and Walker, on pages 1241–3.)**

Enteric infections may contribute to stunted growth in early childhood through clinical diarrhea and through a subclinical condition termed environmental enteric dysfunction (EED), which is characterized by intestinal inflammation, malabsorption, and gut permeability. Observationally, enteric infections have been associated with poor linear growth in a variety of settings [1–5]. A recent multisite study found that *Shigella*, *Campylobacter*, *Giardia*, and enteroaggregative *Escherichia coli* were most consistently associated with lower length-for-age *z* score (LAZ), though there was variability across sites [5].

Given this putative pathway, enteric infections have been a target for interventions to improve child growth in low-resource settings. Water, sanitation, and hygiene (WASH) interventions should plausibly reduce enteropathogen exposure, infection, diarrheal illness, and EED [6–8]. However, most previous WASH intervention studies have focused on diarrhea, growth, or other outcomes [9, 10], without assessment of subclinical enteropathogen carriage. Enteric infection outcomes help elucidate the mechanism of impact of WASH interventions and are also objective measures that reduce the potential for recall bias [11].

Previous WASH intervention trials that have assessed enteric infection outcomes have generally focused on parasites detected by microscopy [12–14], or used serologic assays rather than direct detection of enteropathogens [15–17]. These studies found variable results: WASH interventions reduced *Giardia* infections in rural Bangladesh [13], but did not affect parasite infections in India [14, 18], enteric infection serology in Guatemala [16], or enteric infections in Australia [17].

Because of the bidirectional relationship between enteric infections and malnutrition [19], infant and young child feeding (IYCF) interventions to improve infant dietary nutrient intake may reduce the susceptibility of children to enteric infections. For example, undernutrition has been shown to be a risk factor for *Cryptosporidium* infections [20, 21]. Furthermore, there may be synergy between IYCF and WASH interventions when implemented together, since reduced EED from the WASH intervention may improve uptake and utilization of nutrients from the diet. This synergy may be necessary for major improvements in growth as interventions to improve infant diets alone have had only a modest impact on stunting [22, 23].

The Sanitation Hygiene Infant Nutrition Efficacy (SHINE) Trial was designed to test the independent and combined effects of WASH and IYCF interventions on linear growth in rural Zimbabwe using a cluster-randomized, factorial design. The primary trial results showed that the IYCF intervention had a significant but modest impact on linear growth, but that the WASH intervention, both alone and in combination with the IYCF intervention, did not lead to a significant increase in LAZ at 18 months of age [24]. There are several possible explanations for the lack of impact of the WASH intervention: (1) the interventions did not reduce the enteric infections that affect growth; (2) the interventions reduced the relevant enteric infections but the reduction was not large enough to improve growth; (3) the interventions reduced enteric infections, but the association between enteric infections and growth is not causal. To distinguish between these 3 possibilities, we assessed the impact of the interventions on enteric infections and pathogen-attributable diarrhea among a subset of children in the SHINE trial using highly sensitive quantitative molecular diagnostics for 29 pathogens.

## METHODS

The SHINE trial has been previously described [24, 25]. In brief, SHINE was a 2 × 2 factorial cluster-randomized controlled trial testing the effects of improved WASH and ICYF on stunting and anemia among children at 18 months of age in rural Zimbabwe (described in detail in [25]). The WASH intervention comprised a ventilated improved pit latrine, 2 handwashing stations, monthly delivery of soap and chlorine, a clean play space to separate children from animals and reduce geophagia, and behavior change modules promoting use of these tools. The IYCF intervention included a daily small-quantity lipid-based nutrient supplement (20 g) between 6 and 18 months of age and complementary feeding counseling. Village health workers enrolled women in pregnancy, delivered intervention modules monthly until 12 months postpartum, and provided informal reminders thereafter. A separate research team collected anthropometry from enrolled infants at 1, 3, 6, 12, and 18 months of age. Length measurements were converted into LAZ using 2006 World Health Organization child growth standards [26]. Rotavirus vaccine was introduced to the Zimbabwean immunization schedule partway through the SHINE trial, in 2014.

Among 5280 enrolled women, there were 3989 human immunodeficiency virus (HIV)–unexposed live-born infants. A subsample of 1169 HIV-unexposed infants was enrolled in the EED substudy, which entailed longitudinal collection of biospecimens [27]. Stool samples were collected at 1, 3, 6, 12, and 18 months of age regardless of the report of diarrhea, which was defined by maternal report of ≥3 loose or watery stools in 24 hours or 1 stool with blood or mucus. Among those in the EED substudy, a subsample of 562 HIV-unexposed infants was also enrolled in a diarrhea substudy for whom stool samples were collected during diarrhea. The Medical Research Council of Zimbabwe and the Institutional Review Board of the Johns Hopkins Bloomberg School of Public Health approved the study protocol. Mothers provided written informed consent as well as separate written informed consent for the EED substudy.

We tested all available stool specimens from HIV-unexposed infants in the EED substudy at 1, 3, 6, and 12 months of age and all diarrheal stools up to 18 months from HIV-unexposed infants in the diarrhea substudy with custom-developed TaqMan Array Cards (TACs; ThermoFisher) that compartmentalized probe-based quantitative polymerase chain reaction (qPCR) assays for 29 enteropathogens (Supplementary Table 1) as previously described [5]. Assay validation, nucleic acid extraction, qPCR conditions, and quality control were previously described [28, 29]. In brief, 200 mg of stool was extracted using QIAamp Fast DNA Stool Mini Kit (Qiagen) with 2 external controls (bacteriophage MS2 and phocine herpesvirus). The qPCR assays performed on TAC were set up with the AgPath One Step real-time PCR kit (ThermoFisher). We included one extraction blank per batch and one no-template amplification control per 10 cards to exclude laboratory contamination. Standard curves were generated using positive control constructs, and used to convert qPCR cycle threshold (Cq) values to target copy numbers. We have previously demonstrated that analyses based on Cq values and copy numbers yielded similar results [5, 28]. Pathogen quantities presented here were defined by log_10_-copy numbers per gram of stool based on the Cq. Pathogen quantities for stools in which the pathogen was not detected (Cq ≥ 35) were set at the $\frac{Limit\;of\;detection\;}{2}$ = 1.8495 log_10_-copy numbers per gram of stool. For individual pathogen analyses, we included all 15 pathogens with ≥2% prevalence.

All intervention effects were intention-to-treat at the child level. Initial models included the main effects of the IYCF and WASH interventions and the statistical interaction between the 2 interventions. As in previous prespecified analyses [24], when this interaction term was not statistically significant (*P* > .05), only the main effects were retained. We accounted for multiple comparisons of the 15 enteropathogens in each analysis using the Benjamini–Hochberg procedure [30].

### Data Analysis: Intervention Effects on Enteropathogen Infection

To estimate the impact of the interventions on enteropathogen prevalence, we used linear binomial regression adjusting for age at stool collection and used generalized estimating equations with robust variance to account for correlation within clusters and among children’s outcomes over time. Enteropathogen infection outcomes were dichotomized at each time point with positives defined by Cq < 35 (the analytic limit of detection). Due to the low prevalence of enteropathogen detections at 1 and 3 months, models including stools from all 4 time points did not converge when adjusting for age at sample collection. Therefore, primary analyses included all stools collected during visits at 6 and 12 months of age. We incorporated the 1- and 3-month stools in a sensitivity analysis in which we considered enteropathogen prevalence at each time point individually as outcomes.

We similarly assessed average quantity of detected pathogens, including both positive and negative stools, using a 2-part model (logistic regression for detection and log-normal regression for quantity, given detection) to handle the zero-inflated semicontinuous data. We estimated the marginal differences in absolute quantity between intervention groups using the parametric g-formula and bootstrap with 1000 resamples at the cluster level for confidence intervals (CIs). Specifically, we used Monte Carlo simulations with the estimated β-coefficients from the logistic and log-normal models to predict pathogen detection and quantity under each intervention in a random sample of replicates from the study population 100 times the sample size. We estimated marginal quantity differences by taking the difference of the mean predicted quantities between intervention simulations.

To assess impact on overall pathogen burden, we estimated the difference in number of total pathogens, bacteria, viruses, and parasites detected between intervention groups using Poisson regression and the parametric g-formula as above.

We repeated analyses adjusting for baseline covariates to account for residual confounding and improve precision. We retained all prespecified covariates (Supplementary Table 2) if they were associated with the pathogen outcomes with *P* < .2 in bivariable analyses. We also performed a secondary per-protocol analysis which only included children who received the interventions with high fidelity, defined as receiving all 5 core behavior-change modules for each intervention [25].

### Data Analysis: Intervention Effects on Pathogen-Attributable Diarrhea

To assess the impact of the interventions on pathogen-specific diarrhea, we used the Andersen and Gill extension of the Cox model with age as the timescale to account for multiple diarrhea episodes and variable follow-up time for each child in the diarrhea substudy. We adjusted for month of entry into the diarrhea substudy to account for seasonality. Enteropathogen outcomes during diarrhea were defined by (1) any detection of the pathogen (Cq < 35) and (2) etiologic attribution of the episode to that pathogen. Pathogen-specific etiologic attribution was determined using the adjusted attributable fraction (AFe) for each episode to account for subclinical infections, as previously described [28, 31, 32]. In brief, we used the pathogen quantity, age, and sex-specific odds ratios (ORs) for diarrhea derived from the Global Enteric Multicenter Study (GEMS) and the Etiology, Risk Factors and Interactions of Enteric Infections and Malnutrition and the Consequences for Child Health and Development (MAL-ED) study [28, 31] to estimate the AFe for each diarrhea episode in SHINE as: 1 – 1 / OR. We defined pathogen-attributable episodes when the pathogen quantity-derived AFe ≥ 0.5.

### Data Analysis: Potential Impact of Enteropathogens on Growth

To estimate the expected impact of observed pathogen reductions due to the IYCF or WASH interventions on linear growth, we used longitudinal models previously developed to associate monthly enteropathogen detections with linear growth in the observational MAL-ED study [5]. Using the parametric g-formula, we predicted average LAZ at 24 months among children in MAL-ED under their observed pathogen exposure and under a scenario of reduced pathogen exposure, specifically reduced by the prevalence difference observed due to the WASH and/or IYCF interventions in SHINE. LAZ differences between these 2 scenarios estimate the expected intervention impact in the observational cohort and indicate whether observed pathogen reductions would be expected to be sufficient to improve population-level linear growth.

We also assessed whether the observed enteric infections in SHINE were associated with LAZ outcomes in SHINE, adjusting for WASH and IYCF intervention randomization groups. However, because of the few time points tested and low pathogen prevalence at 1 and 3 months of age, we could only compare linear growth between children with 1 or more pathogen detections to those with no detections. Further details of the methods and results for this analysis are included in the Supplementary Materials.

## RESULTS

For the analysis of enteropathogen infections among stools collected at 1, 3, 6, and 12 months of age, we included 2181 stools from 992 HIV-unexposed children in the EED substudy who had at least 1 stool sample with valid qPCR results. These children generally had similar distributions of baseline household, sanitation, and drinking water characteristics compared to the full trial population. However, they had more access to solar power (73% vs 66%); were more likely to have a handwashing station (12% vs 8%), livestock in the house (41% vs 36%), and feces observed in the yard (34% vs 30%); and had higher diet diversity (47% vs 38% meeting minimum diet diversity score) (Supplementary Table 3). Baseline characteristics of children included in this analysis were generally balanced across intervention arms (Supplementary Table 4). The main trial results in this subset were consistent with the full cohort, though all intervention effects for LAZ in the EED substudy were closer to the null and less precise than those in the full trial.

The majority of stool samples were collected at 6 (n = 721 [33.1%]) and 12 (n = 826 [37.9%]) months of age with the remaining at 1 and 3 months (n = 313 and 321, respectively). Most (n = 933 [94.1%]) children had 6- and/or 12-month samples, 614 (61.9%) children had both 6- and 12-month samples, and 95 (9.6%) children had samples at all 4 time points. Almost all samples collected at scheduled visits were nondiarrheal; only 110 (5.0%) of samples were watery or loose. There was no difference in the prevalence of diarrheal stools across intervention arms: *P* = .2 comparing WASH (n = 52 [5.8%]) vs non-WASH (n = 59 [4.6%]) arms; *P* = .8 comparing IYCF (n = 60 [5.2%]) vs non-IYCF (n = 51 [4.9%]) arms.

**Figure 1.**
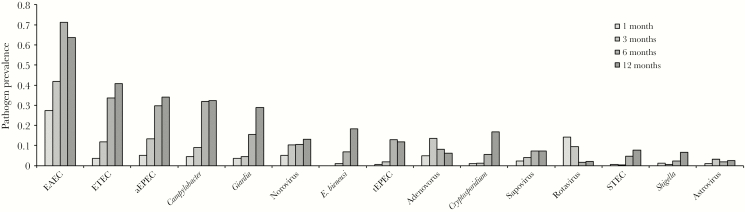
Enteropathogen prevalence in stool samples collected from 996 children in the Sanitation Hygiene Infant Nutrition Efficacy (SHINE) trial. High prevalence of rotavirus at 1 and 3 months of age may be due to shedding of rotavirus vaccine. Abbreviations: aEPEC, atypical enteropathogenic *Escherichia coli*; EAEC, enteroaggregative *Escherichia coli*; ETEC, enterotoxigenic *Escherichia coli*; STEC, Shiga toxin–producing *Escherichia coli*; tEPEC, typical enteropathogenic *Escherichia coli*.

Enteroaggregative *E. coli*, enterotoxigenic *E. coli*, atypical enteropathogenic *E. coli* (aEPEC), and *Campylobacter* species were the most prevalent pathogens (Figure 1). Prevalence increased with age for most pathogens. The mean number of pathogens per sample was 0.8 (standard deviation [SD], 0.89) at 1 month of age and increased to 3.0 (SD, 1.43) at 12 months of age. Bacterial pathogens (mean, 2.0 detected at 12 months [SD, 1.07]) were more common than viral (mean, 0.3 [SD, 0.52]) and parasitic (mean, 0.6 [SD, 0.72]) pathogens.

### Intervention Effects on Enteropathogen Infection

In stools collected at 6 and 12 months of age, the WASH and IYCF interventions had no significant impact on the prevalence of 11 of the 15 pathogens (Table 1). There were no statistically significant interactions between the WASH and IYCF intervention arms at 6 and 12 months of age. Children in the WASH intervention arms had an absolute 3% lower (95% CI, 0–7%) prevalence of *Enterocytozoonbieneusi* and an absolute 3% lower (95% CI, 0–5%) prevalence of adenovirus 40/41 compared to those in the non-WASH arms. Conversely, detection of *Campylobacter* species was an absolute 6% (95% CI, 0–11%) higher and detection of sapovirus was 3% higher (95% CI, 0–6%) among children in the IYCF arms compared to the non-IYCF arms.

**Table 1.** Pathogen Prevalence and Quantity Differences Associated With Water, Sanitation, and Hygiene and Infant and Young Child Feeding Interventions Among 1547 Stool Samples Collected at 6 and 12 Months of Age From 933 Children in the Sanitation Hygiene Infant Nutrition Efficacy (SHINE) Environmental Enteric Dysfunction Substudy

Pathogen	Stools Positive at 6 mo (n = 721), No. (%)	Stools Positive at 12 mo (n = 826), No. (%)	Prevalence Difference^a^ at 6 and 12 mo (95% CI)		Quantity^b^ Difference^a^ at 6 and 12 mo (95% CI)	
			WASH	IYCF	WASH	IYCF
Bacteria						
EAEC	514 (71.3)	526 (63.7)	.01 (–.03 to .06)	–.03 (–.07 to .01)	.03 (–.19 to .23)	–.06 (–.29 to .14)
ETEC	242 (33.6)	336 (40.7)	–.01 (–.07 to .05)	.01 (–.05 to .06)	–.02 (–.24 to .24)	.04 (–.24 to .21)
aEPEC	215 (29.8)	281 (34)	.03 (–.02 to .08)	.03 (–.02 to .08)	.08 (–.10 to .27)	.15 (–.02 to .34)
*Campylobacter* spp	230 (31.9)	267 (32.3)	–.00 (–.06 to .05)	.06 (.00–.11)	.00 (–.17 to .21)	.15 (–.06 to .33)
tEPEC	93 (12.9)	98 (11.9)	–.02 (–.05 to .02)	–.03 (–.07 to .01)	–.06 (–.22 to .08)	–.11 (–.28 to .03)
STEC	33 (4.6)	63 (7.6)	–.00 (–.03 to .02)	–.01 (–.04 to .02)	–.01 (–.09 to .11)	–.04 (–.16 to .04)
*Shigella*	16 (2.2)	55 (6.7)	–.01 (–.03 to .01)	–.01 (–.02 to .01)	.00 (–.11 to .19)	–.07 (–.15 to .05)
Viruses						
Norovirus	76 (10.5)	109 (13.2)	.02 (–.02 to .05)	–.01 (–.05 to .02)	.08 (–.07 to .18)	–.04 (–.13 to .10)
Sapovirus	52 (7.2)	60 (7.3)	–.00 (–.03 to .03)	.03 (.00–.06)	–.01 (–.10 to .08)	.16 (.06–.25)
Adenovirus 40/41	59 (8.2)	52 (6.3)	–.03 (–.05 to –.00)	–.00 (–.03 to .03)	–.07 (–.16 to .03)	–.03 (–.14 to .06)
Astrovirus	13 (1.8)	21 (2.5)	.01 (–.00 to .02)	.01 (–.00 to .02)	.06 (.00–.13)	.05 (–.00 to .11)
Rotavirus	12 (1.7)	17 (2.1)	–.00 (–.02 to .01)	–.00 (–.02 to .01)	.01 (–.03 to .06)	–.01 (–.06 to .03)
Protozoa						
*Giardia*	112 (15.5)	238 (28.8)	–.04 (–.09 to .00)	–.01 (–.06 to .03)	–.16 (–.33 to .03)	–.07 (–.29 to .09)
*Enterocytozoon bieneusi*	49 (6.8)	151 (18.3)	–.03 (–.07 to –.00)	.01 (–.02 to .04)	–.13 (–.29 to –.03)	.01 (–.08 to .17)
*Cryptosporidium*	40 (5.5)	138 (16.7)	.00 (–.030 to .04)	.01 (–.02 to .04)	.10 (–.08 to .15)	.04 (–.06 to .16)

Abbreviations: aEPEC, atypical enteropathogenic *Escherichia coli*; CI, confidence interval; EAEC, enteroaggregative *Escherichia coli*; ETEC, enterotoxigenic *Escherichia coli*; IYCF, infant and young child feeding; STEC, Shiga toxin–producing *Escherichia coli*; tEPEC, typical enteropathogenic *Escherichia coli*; WASH, water, sanitation, and hygiene.

^a^Adjusted for age in days of sample collection.

^b^Quantity measured in log-copy numbers per gram of stool.

There was some variation across time points. Intervention effects were largest at 12 months of age when pathogens were most prevalent (Supplementary Table 5). There was a significant interaction between WASH and IYCF for *Giardia* at 12 months (*P* for heterogeneity = .03), such that there was an absolute 10% reduction (95% CI, 1%–19%) in *Giardia* in the combined WASH + IYCF arm compared to control at 12 months of age, but no reduction in the WASH alone or IYCF alone arms compared to control. There was also a decrease of sapovirus and an increase of astrovirus and atypical EPEC in the WASH arms at 12 months (Supplementary Table 5). With adjustment for multiple comparisons, there was no significant difference in the prevalence of any pathogen at any time point across intervention arms.

Differences in mean quantity of pathogens detected at 6 and 12 months between intervention arms were consistent with prevalence differences (Table 1; Supplementary Table 6). For example, mean quantity of *E. bieneusi* was 0.13 log-copy numbers per gram of stool (95% CI, .03–.29) lower in the WASH arms compared to non-WASH arms at 6 and 12 months.

There were no significant differences in the overall number of pathogens or number of bacteria or viruses detected at 6 and 12 months of age. In contrast, there was a decrease in the number of parasites detected in the WASH arms compared to the non-WASH arms (difference in parasite score, –0.07 [95% CI, –.14 to –.02; Table 2). This difference was driven by reduction in parasites at the 12-month time point (Supplementary Table 7). Adjusted prevalence differences (Supplementary Tables 8 and 9) did not differ substantially from the unadjusted analyses above. Most children received the interventions with high fidelity (WASH: n = 373 [86.9%]; IYCF: n = 451 [85.7%]; control: n = 216 [80.3%]). Prevalence differences among children who received the interventions with high fidelity were similar to those in the full cohort (Supplementary Table 10).

**Table 2.** Differences in Pathogen Group Scores per Stool Sample in Water, Sanitation, and Hygiene (WASH) Versus Non-WASH and Infant and Young Child Feeding (IYCF) Versus Non-IYCF Treatment Arms Among 1547 Stool Samples Collected at 6 and 12 Months of Age From 933 Children in the Sanitation Hygiene Infant Nutrition Efficacy (SHINE) Environmental Enteric Dysfunction Substudy

Pathogen group	Mean (SD) Pathogen Score^a^		Score Difference^b^ (95% CI)	Mean (SD) Pathogen Score^a^		Score Difference^b^ (95% CI)
	WASH	Non-WASH	WASH vs Non-WASH	IYCF	Non-IYCF	IYCF vs Non-IYCF
All pathogens	2.8 (1.46)	2.8 (1.46)	–.06 (–.22 to .06)	2.8 (1.46)	2.8 (1.46)	–.01 (–.17 to .12)
Bacteria	2.0 (1.16)	2.0 (1.09)	.02 (–.10 to .12)	2.0 (1.16)	2.0 (1.09)	–.02 (–.15 to .07)
Viruses	0.3 (0.51)	0.3 (0.53)	–.00 (–.06 to .05)	0.3 (0.51)	0.3 (0.53)	.02 (–.03 to .07)
Parasites	0.4 (0.63)	0.5 (0.69)	–.07 (–.14 to –.02)	0.4 (0.63)	0.5 (0.69)	.00 (–.06 to .06)

Abbreviations: CI, confidence interval; IYCF, infant and young child feeding; SD, standard deviation; WASH, water, sanitation, and hygiene.

^a^Number of pathogens in group detected per stool sample among stools collected at 6 and 12 months of age.

^b^Adjusted for age in days of sample collection.

### Intervention Effects on Pathogen-Attributable Diarrhea

Five hundred sixty-two HIV-unexposed children in the diarrhea substudy were followed for the collection of diarrheal stools for a median of 14.4 months (interquartile range [IQR], 13.5–14.8), during which 161 of 527 reported episodes (30.6%) had stool samples collected and validly tested by qPCR. The proportions of episodes with samples collected were similar across intervention arms (31.1% in WASH arms, 30.3% in non-WASH arms, 30.0% in IYCF arms, and 31.4% in non-IYCF arms). At least 1 enteric pathogen was detected in 94.4% of episodes and the median number of pathogens detected was 3 (IQR, 2–4). However, only 47.2% of episodes could be attributed etiologically to a pathogen (ie, the pathogen quantity detected was high enough to be associated with diarrhea). There were no significant differences in the number of pathogens detected or attributed across intervention arms.

There were also no statistically significant differences in the detection or attribution of specific pathogens (Table 3). The rate of *Shigella*-attributable diarrhea was lower among children in the IYCF arms compared to the non-IYCF arms (hazard ratio, 0.23 [95% CI, .07–.77]), but this estimate was based on few attributable episodes (n = 13) and was not significant after adjustment for multiple comparisons.

**Table 3.** Differences in Rates of Pathogen-Attributable Diarrhea Associated With Water, Sanitation, and Hygiene and Infant and Young Child Feeding Interventions Among 161 Diarrhea Episodes From 129 Children in the Sanitation Hygiene Infant Nutrition Efficacy (SHINE) Diarrhea Substudy

	Pathogen-Attributable Episodes (n = 159^a^), No. (%)	Episodes With Pathogen Detected (n = 161), No. (%)	Pathogen-Attributable Diarrhea, HR^b^ (95% CI)		Pathogen Detected During Diarrhea, HR^b^ (95% CI)	
Pathogen			WASH	IYCF	WASH	IYCF
Bacteria						
EAEC	0	92 (56.8)	…	…	0.87 (.55–1.36)	1.00 (.64–1.57)
ETEC	12^c^ (7.6)	59 (37.9)	3.18 (.80–12.71)	2.28 (.60–8.71)	1.03 (.61–1.74)	1.13 (.67–1.90)
aEPEC	0	45 (27.8)	…	…	.77 (.41–1.45)	1.40 (.79–2.49)
*Campylobacter* spp	2^c^ (1.3)	62 (38.3)	…	…	1.13 (.63–2.03)	1.21 (.69–2.15)
tEPEC	1 (0.6)	12 (7.4)	…	…	.63 (.19–2.10)	.56 (.17–1.91)
STEC	0	7 (4.4)	…	…	.27 (.03–2.32)	.59 (.11–3.08)
*Shigella*	13 (8.2)	20 (12.4)	.60 (.16–2.21)	.23 (.07–.77)	.50 (.17–1.47)	.33 (.10–1.11)
Viruses						
Norovirus	15^c^ (9.4)	44 (27.3)	1.47 (.51–4.23)	1.15 (.42–3.12)	.91 (.47–1.74)	1.48 (.83–2.66)
Sapovirus	11 (6.9)	23 (14.2)	1.92 (.58–6.41)	1.34 (.36–4.96)	1.03 (.43–2.45)	1.75 (.70–4.36)
Adenovirus 40/41	4 (2.5)	28 (17.3)	.53 (.06–4.69)	2.48 (.25–24.60)	1.12 (.53–2.35)	.88 (.41–1.87)
Astrovirus	5 (3.1)	17 (10.5)	1.38 (.22–8.62)	.19 (.02–1.47)	1.16 (.44–3.07)	.89 (.34–2.35)
Rotavirus	12 (7.6)	19 (11.7)	.79 (.25–2.55)	1.08 (.34–3.40)	.96 (.39–2.34)	1.05 (.43–2.58)
Protozoa						
*Giardia*	0	26 (16.0)	…	…	.49 (.18–1.32)	1.25 (.54–2.92)
*Enterocytozoon bieneusi*	0	26 (16.3)	…	…	.53 (.22–1.27)	1.25 (.55–2.85)
*Cryptosporidium*	12 (7.6)	42 (25.9)	2.41 (.81–7.23)	.54 (.18–1.65)	.83 (.42–1.63)	.95 (.49–1.85)

Abbreviations: aEPEC, atypical enteropathogenic *Escherichia coli*; CI, confidence interval; EAEC, enteroaggregative *Escherichia coli*; ETEC, enterotoxigenic *Escherichia coli*; HR, hazard ratio; IYCF, infant and young child feeding; STEC, Shiga toxin–producing *Escherichia coli*; tEPEC, typical enteropathogenic *Escherichia coli*; WASH, water, sanitation, and hygiene.

^a^Excludes 3 episodes for which valid quantitative polymerase chain reaction results were not available for all pathogens included in the attribution analysis.

^b^Adjusted for calendar month at the start of diarrhea surveillance.

^c^All ETEC-attributable episodes were heat-stable enterotoxin-producing ETEC (ST-ETEC); all *Campylobacter*-attributable episodes were *Campylobacter jejuni/coli*; all norovirus-attributable episodes were norovirus GII.

### Potential Impact of Enteropathogens on Growth

Using the longitudinal growth models from the observational MAL-ED study, we estimated the expected linear growth impact of the largest pathogen reductions observed in SHINE, which were an absolute 10% reduction in *Giardia* prevalence and a 6% reduction in *E. bieneusi* prevalence at 12 months of age (Supplementary Table 5). The estimated improvement in mean LAZ at 24 months that would be expected due to a 10% reduction in *Giardia* prevalence from 12 to 24 months of age was 0.02 LAZ (95% CI, .00–.04). Similarly, the estimated improvement in mean LAZ at 24 months that would be expected based on a 6% reduction in *E. bieneusi* prevalence from 12 to 24 months of age was 0.02 LAZ (95% CI, .00–.04).

Observational associations between enteropathogen detections and LAZ differences at 12 and 18 months in SHINE were small and imprecise. Strongest associations between exposures from 1 to 12 months of age and linear growth were with LAZ measured at 12 months (Supplementary Tables 11 and 12).

## DISCUSSION

Enteric infections were common among children in the first year of life in rural Zimbabwe. The WASH interventions modestly decreased the prevalence of total parasites but had no apparent impact on bacterial or viral infections. The IYCF interventions did not impact enteropathogen infections. For both enteric infections and pathogen-attributable diarrhea, WASH and IYCF intervention effects for individual pathogens were small and were not statistically significant after accounting for multiple comparisons. There was also no evidence of synergy between the WASH and IYCF interventions, except potentially for *Giardia* at 12 months of age. The reduction of parasites with WASH interventions has been noted previously in a similar WASH and nutritional intervention trial conducted in rural Bangladesh. Specifically, handwashing and sanitation interventions reduced *Giardia* prevalence at 2.5 years of age, though chlorinated drinking water alone and nutrition improvements had no effect [13].

The longitudinal growth models from an observational study, MAL-ED, predict that the small and inconsistent pathogen prevalence reductions achieved by the interventions in SHINE would not translate to substantial improvements in average linear growth outcomes (<0.05 LAZ). Therefore, the null effects of the WASH interventions on linear growth [24] may be explained at least in part by the failure of the interventions to prevent enteric infections.

Because the interventions did not substantially reduce the burden of enteropathogens, the hypothesis that enteropathogens inhibit linear growth remains untested. The observational associations between enteropathogens and linear growth in SHINE were variable and imprecise, likely because of the infrequent sampling only in the first year of life. Randomized studies evaluating WASH interventions that are more efficacious in reducing enteric infections than those delivered in SHINE will better test the hypothesis that enteropathogens cause poor linear growth among children in low-resource settings.

This analysis was limited by the relatively few sampling time points to capture enteric infections. Specifically, qPCR testing was not performed on the 18-month stool samples collected in SHINE such that no exposures were captured in the second year of life when bacterial and parasitic pathogens are more common [5]. However, because the latrines were delivered during pregnancy, and the WASH behavior change modules were all delivered by 12 months of age, we would have expected to see an effect by 12 months. The analysis was also limited by a relatively low proportion of diarrhea episodes having stools collected. However, collection rates did not differ across intervention arms and we accounted for variable ages and durations of diarrhea surveillance periods, such that underdetection should not bias comparisons across groups.

In sum, the elementary household-level WASH interventions tested in SHINE did not meaningfully reduce fecal–oral microbial transmission in children during the first year of life. Transformative WASH interventions that are more efficacious in interrupting fecal exposure among young children living in highly contaminated environments are needed. Such transformative approaches may include improved technologies, more intense behavior change strategies, and stronger governance of the human systems implementing these interventions.

## Supplementary Data

Supplementary materials are available at *The Journal of Infectious Diseases* online. Consisting of data provided by the authors to benefit the reader, the posted materials are not copyedited and are the sole responsibility of the authors, so questions or comments should be addressed to the corresponding author.

jiz179_Suppl_Supplementary_Material

## Notes

***Financial support.*** The SHINE trial is funded by the Bill & Melinda Gates Foundation (grant numbers OPP1021542 and OPP1143707); the UK Department for International Development; the Wellcome Trust, United Kingdom (grant numbers 093768/Z/10/Z, 108065/Z/15/Z, and 201293/Z/16/Z); the Swiss Agency for Development and Cooperation ; the US National Institutes of Health (grant number 2R01HD060338-06); and the European Union. This analysis was further supported by the US National Institutes of Health (grant number K01AI130326 to E. T. R. M.).

***Potential conflicts of interest.*** All authors: No reported conflicts of interest. All authors have submitted the ICMJE Form for Disclosure of Potential Conflicts of Interest. Conflicts that the editors consider relevant to the content of the manuscript have been disclosed.
