# Supplementary material for: Impact of Water Quality, Sanitation, Handwashing, and Nutritional Interventions on Enteric Infections in Rural Zimbabwe: The Sanitation Hygiene Infant Nutrition Efficacy (SHINE) Trial
Source: J Infect Dis. 2019 Apr 20;221(8):1379–86. doi: 10.1093/infdis/jiz179 (PMC7325799; doi:10.1093/infdis/jiz179)
Supplement: jiz179_Suppl_Supplementary_Material [file jiz179_suppl_supplementary_material.pdf]

Supplemental material

Impact of water quality, sanitation, handwashing, and nutritional interventions on enteric infections in rural Zimbabwe: the Sanitation Hygiene Infant Nutrition Efficacy (SHINE) Trial

Elizabeth T. Rogawski McQuade[1,2], James A. Platts-Mills[2], Jean Gratz[2], Jixian Zhang[2], Lawrence H. Moulton[3,4], Kuda Mutasa[3], Florence D. Majo[3], Naume Tavengwa[3], Robert Ntozini[3], Andrew J. Prendergast[3,5], Jean H. Humphrey[3,4], Jie Liu[2], Eric R. Houpt[2]

[1]Department of Public Health Sciences, University of Virginia, Charlottesville, VA 22908, USA
[2]Division of Infectious Diseases & International Health, University of Virginia, Charlottesville, VA 22908, USA
[3]Zvitambo Institute for Maternal and Child Health Research, Harare, Zimbabwe
[4]Department of International Health, Johns Hopkins Bloomberg School of Public Health, Baltimore, MD 21205, USA
[5]Blizard Institute, Queen Mary University of London, United Kingdom

Table S1. Real time PCR assays on TaqMan Array Card used in SHINE.

All the assays have been described previously and extensively validated [1–3]. Nucleic acid was extracted with the QIAamp Fast DNA Stool mini kit (Qiagen, Hilden, Germany) with pre-treatment steps that included bead beating. AgPath One Step RT-PCR reagents were used for qPCR reactions, which were performed on ViiA 7 systems. Quantification cycles (Cqs) are the PCR cycle values at which fluorescence from amplification exceeds the background, which acts as an inverse metric of quantity of nucleic acid. Valid results required proper functioning of controls (the negative results of a sample are valid only when its external control MS2 is positive, Cq ≤ 35; the positive results are valid only when the corresponding extraction blank is negative for the relevant targets, Cq > 35), and excluded data flagged by the real time PCR software, i.e. BADROX in combination with NOISE or SPIKE.

	Pathogen	Gene
Viruses	Adenovirus 40/41	Fiber gene
	Astrovirus	Capsid
	Norovirus GI/GII	GI ORF1-2 and GII ORF1-2
	Rotavirus	*NSP3*
	Sapovirus	*RdRp*
Bacteria	EAEC*	*aaiC, aatA, aggR*
	Atypical EPEC*	*eae*
	Typical EPEC*	*bfpA*
	ETEC*	*LT, STh* and *STp*
	STEC*	*stx1, stx2*
	Aeromonas	Aerolysin
	Campylobacter spp.	*cadF* and *cpn60*
	Helicobacter pylori	*ureC*
	Plesiomonas shigelloides	*gyrB*
	Salmonella	*ttr*
	Shigella/EIEC	*ipaH*
	Vibrio cholerae	*hlyA*
Fungi	*Enterocytozoon bieneusi*	*ITS*
	Encephalitozoon intestinalis	SSU rRNA
Protozoa	*Cryptosporidium*	18S rRNA
	Cyclospora cayetanensis	18S rRNA
	Cystoisospora belli	18S rRNA
	Entamoeba histolytica	18S rRNA
	Giardia	18S rRNA
Helminth	*Ancylostoma duodenale*	*ITS2*
	Ascaris lumbricoides	*ITS1*
	Necator americanus	*ITS2*
	Strongyloides stercoralis	Dispersed repetitive sequence
	Trichuris trichiura	18S rRNA
Controls	MS2	*MS2g1*
	PhHV	*gB*

**E. coli* pathotypes were defined as follows: EAEC (*aaiC*, or *aatA*, or both), atypical EPEC (*eae* without *bfpA*, *stx1*, and *stx2*), typical EPEC (*bfpA*), ETEC (STh, STp, or LT), STEC (*eae* without *bfpA* and with *stx1*, *stx2*, or both).

Table S2. Covariates considered in adjusted analyses.

Category	Variable
Maternal	Age
	Height
	MUAC
	Years of completed schooling
	Marital status
	Parity
	Employment status
	Religion
	Maternal depression (Edinburgh Postnatal Depression Score; EPDS)
	Maternal hemoglobin
Maternal capabilities	Decision making autonomy
	Gender norms and attitudes
	Perceived social support
	Perceived physical health
	Mothering self-efficacy
	Perceived time stress
Household	Household Coping Strategies Index
	Any latrine
	Improved latrine
	Open defecation
	Feces observed in yard
	Improved floor
	Time to drinking water
	Water treatment
	Owns chickens
	Livestock observed in house
	Household size
	Wealth quintile
Infant	Birth length
	Low birthweight (<2500 g)
	Gender
	Preterm birth
	Loose, watery, or bloody stool
Study	Calendar quarter of the study

Table S3. Comparison of maternal, household, and infant characteristics of HIV-negative mothers of children included and excluded from the analysis of stool samples tested by qPCR.

Baseline Characteristic	Included (Mothers: N=980; Children: N=992)	Excluded (Mothers: N=2957; Children: N=2997)	p-value
Baseline visit completed, n (%)	945 (96.43%)	2744 (92.80%)	
Household characteristics			
Household size median (IQR), [n]	5 (4,6) [965]	5 (3,6) [2821]	0.7
Wealth Quintile, n/N (%):			0.2
Lowest	158/941 (16.79%)	522/2718 (19.21%)	
Second	198/941 (21.04%)	508/2718 (18.69%)	
Middle	203/941 (21.57%)	540/2718 (19.87%)	
Fourth	197/941 (20.94%)	570/2718 (20.97%)	
Highest	185/941 (19.66%)	578/2718 (21.27%)	
Electricity, n/N (%)	25/941 (2.66%)	75/2715 (2.76%)	0.9
Other electric power, n/N (%):			<0.001
Generator	24/941 (2.55%)	94/2716 (3.46%)	
Solar power	684/941 (72.69%)	1799/2716 (66.24%)	
None/other	233/941 (24.76%)	823/2716 (30.30%)	
Sanitation			
Open defecation, n/N* (%)	2200/4421 (49.76%)	5482/11444 (47.90%)	0.01
Among aged 0 – 2.9 years	170/289 (58.82%)	430/774 (55.56%)	0.02
Among aged 3 – 5.9 years	261/471 (55.41%)	587/1062 (55.27%)	0.2
Among aged 6 – 17.9 years	607/1144 (53.06%)	1239/2662 (46.54%)	0.003
Among aged 18 – 69.9 years	945/1975 (47.85%)	2378/4998 (47.58%)	0.02
Among aged 70+ years	19/47 (40.43%)	38/129 (29.46%)	0.1
Any latrine, n/N (%)	322/921 (34.96%)	1002/2685 (37.32%)	0.2
Improved latrine	280/920 (30.43%)	877/2681 (32.71%)	0.2
Improved latrine with well- trodden path	250/920 (27.17%)	768/2681 (28.65%)	0.4
Improved latrine with well-trodden path and not shared	217/891 (24.35%)	673/2593 (25.95%)	0.3
Water			
Improved water, n/N (%)	575/924 (62.23%)	1718/2701 (63.61%)	0.5
Treat water, n/N (%)	112/912 (12.28%)	337/2656 (12.69%)	0.7
Time to collect drinking water (min), median (IQR) [n]	10 (5,15) [922]	10 (5,20) [2693]	<0.001
Per capita water volume, mean (SD)	9.34 (8.07)	9.77 (11.58)	<0.001
Hygiene			
Handwashing station present, n/N (%)	106/907 (11.69%)	202/2526 (8.00%)	0.001

Handwashing with water, n/N (%)	35/905 (3.87%)	76/2522 (3.01%)	0.2
Improved floor, n/N (%)	513/929 (55.22%)	1483/2678 (55.38%)	0.9
No. chickens, median (IQR), (n)	6 (2,11) [944]	6 (2,10) [2698]	0.2
Livestock in house, n/N (%)	386/942 (40.98%)	987/2712 (36.39%)	<0.001
Feces observed in yard, n/N (%)	320/939 (34.08%)	816/2696 (30.27%)	0.05
Diet Quality and food security			
Household meets minimum dietary diversity score, n/N (%)	394/845 (46.63%)	899/2379 (37.79%)	<0.001
Coping Strategy Index median (IQR), [n]	0 (0,5) [923]	1 (0,7) [2644]	<0.001
Maternal characteristics			
Age (years), mean (SD) [n]	26.87 (7.73) [926]	25.23 (7.53) [2603]	<0.001
Height (cm), mean (SD) [n]	159.97 (7.92) [957]	159.71 (7.76) [2875]	<0.001
MUAC (cm), mean (SD) [n]	26.75 (3.48) [970]	26.3 (3.14) [2906]	<0.001
S. haematobium positive, n/N (%)	80/922 (8.68%)	298/2558 (11.65%)	0.08
Years of school completed, mean (SD) [n]	9.550 (1.99) [957]	9.6 (2.23) [2786]	<0.001
Parity, median (IQR)	2 (1, 3) [872]	2 (1, 3) [1775]	<0.001
Married, n/N (%)	906/952 (95.17%)	2640/2765 (95.48%)	0.7
Employed, n/N (%)	92/942(9.77%)	219/2714(8.07%)	<0.001
Religion, n/N (%):			0.04
Apostolic	464/958 (48.43%)	1299/2787 (46.61%)	
Other Christian	436/958 (45.51%)	1248/2787 (44.78%)	
Other	58/958 (6.05%)	240/2787 (8.60%)	
Infant characteristics			
Female, n/N (%)	487/992 (49.09%)	1475/2982 (49.46%)	0.8
Birthweight (g), mean (SD) [n]	3.13 (0.5) [962]	3.09 (0.54) [2611]	<0.001
Low birthweight (<2500 g), n/N (%)	77/962 (8.0%)	248/2611 (9.5%)	0.3
Institutional delivery, n/N (%)	859/948 (90.61%)	2302/2608 (88.27%)	0.05
Vaginal delivery, n/N (%)	900/960 (93.75%)	2456/2655 (92.5%)	0.2

*Proportion of individuals practicing open defecation is calculated among all household members, N

Table S4. Maternal, household, and infant characteristics of HIV-negative mothers of children included in the analysis of stool samples tested by qPCR.

Baseline Characteristic	Control (Mothers: N=265; Children: N=269)	WASH (Mothers: N=194; Children: N=197)	IYCF (Mothers: N=292; Children: N=294)	WASH + IYCF (Mothers: N=229; Children: N=232)
Baseline visit completed, n (%)	248 (93.58%)	190 (97.94%)	284 (97.26%)	223 (97.38%)
Household characteristics				
Household size median (IQR), [n]	5 (3,6) [260]	5 (4,6) [190]	5 (4,6) [288]	4 (4,6) [227]
Wealth Quintile, n/N (%):				
Lowest	57/247 (23.08%)	33/190 (17.37%)	34/283 (12.01%)	34/221 (15.38%)
Second	52/247 (21.05%)	38/190 (20.00%)	56/283 (19.79%)	52/221 (23.53%)
Middle	50/247 (20.24%)	46/190 (24.21%)	59/283 (20.85%)	48/221 (21.72%)
Fourth	48/247 (19.43%)	43/190 (22.63%)	66/283 (23.32%)	40/221 (18.10%)
Highest	40/247 (16.19%)	30/190 (15.79%)	68/283 (24.03%)	47/221 (21.27%)
Electricity, n/N (%)	8/246 (3.25%)	5/190 (2.63%)	11/282 (3.9%)	1/223 (0.45%)
Other electric power, n/N (%):				
Generator	4/246 (1.63%)	9/190 (4.74%)	6/282 (2.13%)	5/223 (2.24%)
Solar power	163/246 (66.26%)	138/190 (72.63%)	213/282 (75.53%)	170/223 (76.23%)
None/other	79/246 (32.11%)	43/190 (22.63%)	63/282 (22.34%)	48/223 (21.52%)
Sanitation				
Open defecation, n/N* (%)	623/1071 (58.17%)	415/890 (46.63%)	681/1401 (48.61%)	481/1059 (45.42%)
Among aged 0 – 2.9 years	52/71 (73.24%)	27/50 (54.0%)	49/87 (56.32%)	42/81 (51.85%)
Among aged 3 – 5.9 years	80/116 (68.97%)	51/106 (48.11%)	77/133 (57.89%)	53/116 (45.69%)
Among aged 6 – 17.9 years	162/258 (62.79%)	119/247 (48.18%)	184/349 (52.72%)	142/290 (48.97%)
Among aged 18 – 69.9 years	264/481 (54.89%)	169/397 (42.57%)	293/625 (46.88%)	219/472 (46.4%)
Among aged 70+ years	4/13 (30.77%)	5/10 (50.0%)	5/14 (35.71%)	5/10 (50.0%)
Any latrine, n/N (%)	75/244 (30.74 %	64/182 (35.16%)	114/280 (40.71%)	69/215 (32.09%)
Improved latrine	63/244 (25.82%)	59/181 (32.6%)	97/280 (34.64%)	61/215 (28.37%)
Improved latrine with well- trodden path	54/244 (22.13%)	54/181 (29.83%)	87/280 (31.07%)	55/215 (25.58%)

Improved latrine with well-trodden path and not shared	44/235 (18.72%)	46/175 (26.29%)	75/269 (27.88%)	52/212 (24.53%)
Water				
Improved water, n/N (%)	144/244 (59.02%)	119/184 (64.67%)	179/280 (63.93%)	133/216 (61.57%)
Treat water, n/N (%)	30/240 (12.50%)	24/182 (13.19%)	32/276 (11.59%)	26/214 (12.15%)
Time to collect drinking water (min), median (IQR) [n]	10 (5, 15) [244]	10 (5, 20) [184]	5 (3, 15) [279]	10 (5, 15) [215]
Per capita water volume, mean (SD)	9 (7.28)	8.18 (5.3)	9.26 (5.350)	10.27 (9.880)
Hygiene				
Handwashing station present, n/N (%)	6/232 (2.59%)	42/186 (22.58%)	6/274 (2.19%)	52/215 (24.19%)
Handwashing with water, n/N (%)	2/232 (0.86%)	13/186 (6.99%)	1/274 (0.36%)	19/213 (8.92%)
Improved floor, n/N (%)	118/244 (48.36%)	115/189 (60.85%)	165/279 (59.14%)	115/217 (53%)
No. chickens, median (IQR), (n)	6 (2,10) [249]	6 (2,10) [190]	7 (3,12) [283]	6 (2,10) [222]
Livestock in house, n/N (%)	101/247 (40.89%)	88/190 (46.32%)	108/283 (38.16%)	89/222 (40.09%)
Feces observed in yard, n/N (%)	74/246 (30.08%)	71/190 (37.37%)	104/282 (36.88%)	71/221 (32.13%)
Diet Quality and food security				
Household meets minimum dietary diversity score, n/N (%)	98/225 (43.56%)	82/173 (47.4%)	124/250 (49.6%)	90/197 (45.69%)
Coping Strategy Index median (IQR), [n]	0 (0,5) [246]	0 (0,8) [184]	0 (0,4) [276]	0 (0,4) [217]
Maternal characteristics				
Age (years), mean (SD) [n]	26.37 (7.51) [243]	27.46 (6.39) [185]	26.54 (7.67) [278]	27.38 (8.52) [220]
Height (cm), mean (SD) [n]	159.7 (7.31) [258]	159.4 (8.57) [190]	160.8 (5.78) [286]	159.7 (9.29) [223]
MUAC (cm), mean (SD) [n]	26.43 (2.76) [259]	26.97 (3.59) [194]	26.79 (3.46) [289]	26.79 (3.7) [228]
S. haematobium positive, n/N (%)	14/243 (5.76%)	22/184 (11.96%)	29/276 (10.51%)	15/219 (6.850%)
Years of school completed, mean (SD) [n]	9.52 (1.91) [260]	9.43 (1.91) [188]	9.67 (2.27) [285]	9.55 (2.01) [224]
Parity, median (IQR)	2 (1, 3) [236]	2 (1, 3) [171]	2 (1, 3) [261]	2 (1, 3) [204]

Married, n/N (%)	247/259 (95.37%)	173/188 (92.02%)	269/281 (95.73%)	217/224 (96.88%)
Employed, n/N (%)	15/247 (6.07%)	29/190 (15.26%)	28/282 (9.93%)	20/223 (8.970%)
Religion, n/N (%):				
Apostolic	140/260 (53.85%)	89/189 (47.09%)	127/284 (44.72%)	108/225 (48%)
Other Christian	111/260 (42.69%)	87/189 (46.03%)	142/284 (50%)	96/225 (42.67%)
Other	9/260 (3.46%)	13/189 (6.88%)	15/284 (5.28%)	21/225 (9.33%)
Infant characteristics				
Female, n/N (%)	119/269 (44.24%)	103/197 (52.28%)	145/294 (49.32%)	120/232 (51.72%)
Birthweight (g), mean (SD) [n]	3.11 (0.53) [260]	3.14 (0.40) [189]	3.15 (0.47) [287]	3.12 (0.54) [226]
Low birthweight (<2500 g), n/N (%)	28/260 (10.77%)	14/189 (7.41%)	14/287 (4.88%)	21/226 (9.290%)
Institutional delivery, n/N (%)	234/258 (90.7%)	169/185 (91.35%)	256/284 (90.14%)	200/221 (90.5%)
Vaginal delivery, n/N (%)	247/259 (95.37%)	176/191 (92.15%)	265/285 (92.98%)	212/225 (94.22%)

*Proportion of individuals practicing open defecation is calculated among all household members, N

Table S5. Age-specific pathogen prevalence differences associated with WASH and IYCF interventions compared to control among 2,181 stool samples from 992 children in the SHINE EED substudy.

		1 & 3 months (N=634)			6 months (N=721)		
		Stools positive	Prevalence difference* (95% CI)		Stools positive	Prevalence difference* (95% CI)	
	Pathogen	n (%)	WASH	IYCF	n (%)	WASH	IYCF
Bacteria	EAEC	219 (34.7)	0.03 (-0.05, 0.11)	-0.06 (-0.14, 0.02)	514 (71.3)	0.01 (-0.07, 0.08)	-0.05 (-0.12, 0.02)
	ETEC	49 (7.8)	0.02 (-0.02, 0.07)	0.01 (-0.03, 0.05)	242 (33.6)	-0.04 (-0.11, 0.04)	0.02 (-0.06, 0.09)
	aEPEC	59 (9.3)	-0.00 (-0.05, 0.05)	-0.01 (-0.07, 0.04)	215 (29.8)	0.05 (-0.02, 0.12)	0.06 (-0.00, 0.13)
	Campylobacter spp.	43 (6.8)	-0.01 (-0.05, 0.03)	-0.02 (-0.06, 0.02)	230 (31.9)	0.02 (-0.05, 0.09)	0.04 (-0.04, 0.11)
	tEPEC	8 (1.3)	-0.00 (-0.02, 0.01)	-0.00 (-0.02, 0.01)	93 (12.9)	-0.02 (-0.08, 0.03)	-0.02 (-0.08, 0.04)
	STEC	3 (0.5)	0.01 (-0.01, 0.02)	0.00 (-0.01, 0.02)	33 (4.6)	-0.01 (-0.04, 0.02)	-0.00 (-0.04, 0.03)
	Shigella	6 (0.9)	0.02 (-0.00, 0.04)	0.00 (-0.01, 0.02)	16 (2.2)	-0.02 (-0.03, 0.00)	0.00 (-0.02, 0.02)
Viruses	Norovirus	49 (7.8)	0.02 (-0.03, 0.06)	-0.00 (-0.04, 0.04)	76 (10.5)	0.01 (-0.04, 0.07)	-0.01 (-0.05, 0.04)
	Sapovirus	20 (3.2)	-0.01 (-0.04, 0.02)	-0.02 (-0.05, 0.01)	52 (7.2)	0.01 (-0.03, 0.05)	0.04 (0.01, 0.08)
	Adenovirus 40/41	58 (9.2)	0.00 (-0.04, 0.04)	0.02 (-0.02, 0.06)	59 (8.2)	-0.05 (-0.09, -0.01)	-0.01 (-0.05, 0.04)
	Astrovirus	13 (2.1)	-0.00 (-0.02, 0.02)	0.01 (-0.01, 0.03)	13 (1.8)	-0.01 (-0.03, 0.00)	0.01 (-0.01, 0.03)
	Rotavirus	74 (11.7)	-0.03 (-0.08, 0.02)	-0.01 (-0.05, 0.04)	12 (1.7)	0.00 (-0.02, 0.02)	-0.00 (-0.02, 0.02)
Protozoa	*Giardia*	25 (4.0)	-0.01 (-0.03, 0.02)	-0.00 (-0.03, 0.03)	112 (15.5)	0.01 (-0.04, 0.06)	-0.01 (-0.06, 0.03)
	E. bieneusi	3 (0.5)	-0.00 (-0.01, 0.01)	-0.00 (-0.01, 0.01)	49 (6.8)	-0.01 (-0.04, 0.03)	0.01 (-0.02, 0.05)
	Cryptosporidium	7 (1.1)	0.00 (-0.01, 0.02)	-0.00 (-0.02, 0.01)	40 (5.5)	0.03 (-0.00, 0.07)	0.01 (-0.02, 0.04)

Table S5 continued.

		12 months (n=826)				
		Stools positive	Prevalence difference* (95% CI)			
	Pathogen	n (%)	WASH	IYCF	WASH + IYCF	*p* for heterogeneity
Bacteria	EAEC	526 (63.7)	0.01 (-0.05, 0.07)	-0.01 (-0.07, 0.05)		
	ETEC	336 (40.7)	0.01 (-0.06, 0.09)	-0.01 (-0.08, 0.07)		
	aEPEC	281 (34)	0.09 (0.01, 0.17)	0.06 (-0.02, 0.14)	-0.00 (-0.10, 0.09)	0.04
	Campylobacter spp.	267 (32.3)	-0.03 (-0.10, 0.04)	0.08 (0.00, 0.15)		
	tEPEC	98 (11.9)	-0.02 (-0.07, 0.03)	-0.04 (-0.09, 0.01)		
	STEC	63 (7.6)	-0.00 (-0.04, 0.04)	-0.01 (-0.05, 0.02)		
	Shigella	55 (6.7)	-0.00 (-0.04, 0.03)	-0.01 (-0.05, 0.02)		
Viruses	Norovirus	109 (13.2)	0.02 (-0.03, 0.08)	-0.02 (-0.07, 0.03)		
	Sapovirus	60 (7.3)	-0.05 (-0.10, -0.01)	-0.02 (-0.07, 0.03)	0.01 (-0.05, 0.07)	0.04
	Adenovirus 40/41	52 (6.3)	-0.01 (-0.04, 0.03)	0.01 (-0.03, 0.04)		
	Astrovirus	21 (2.5)	0.03 (0.01, 0.05)	0.01 (-0.01, 0.03)		
	Rotavirus	17 (2.1)	-0.00 (-0.02, 0.02)	-0.01 (-0.03, 0.01)		
Protozoa	*Giardia*	238 (28.8)	-0.01 (-0.09, 0.08)	0.05 (-0.03, 0.13)	-0.10 (-0.19, -0.01)	0.03
	E. bieneusi	151 (18.3)	-0.06 (-0.11, -0.00)	-0.00 (-0.05, 0.05)		
	Cryptosporidium	138 (16.7)	-0.02 (-0.07, 0.03)	0.01 (-0.04, 0.06)		

*Adjusted for age in days of sample collection

Table S6. Age-specific pathogen quantity differences associated with WASH and IYCF interventions compared to control among 2,181 stool samples from 992 children in the SHINE EED substudy.

		1 & 3 months (n=634)			6 months (n=721)		
		Quantity*	Quantity difference† (95% CI)		Quantity*	Quantity difference† (95% CI)	
	Pathogen	Mean (SD)	WASH	IYCF	Mean (SD)	WASH	IYCF
Bacteria	EAEC	6.58 (1.55)	0.17 (-0.20, 0.54)	-0.22 (-0.62, 0.14)	6.61 (1.50)	-0.05 (-0.40, 0.29)	-0.11 (-0.45, 0.24)
	ETEC	5.43 (1.51)	0.09 (-0.18, 0.35)	-0.01 (-0.49, 0.32)	5.94 (1.73)	-0.22 (-0.44, 0.21)	0.08 (-0.32, 0.31)
	aEPEC	5.69 (1.15)	-0.02 (-0.21, 0.27)	-0.07 (-0.24, 0.17)	5.91 (1.35)	0.17 (-0.11, 0.47)	0.43 (0.14, 0.69)
	Campylobacter spp.	5.90 (1.61)	0.04 (-0.16, 0.20)	-0.05 (-0.24, 0.13)	6.17 (1.50)	0.06 (-0.23, 0.36)	-0.02 (-0.37, 0.25)
	tEPEC	6.42 (1.48)	0.01 (-0.11, 0.17)	-0.02 (-0.24, 0.10)	6.41 (1.47)	-0.14 (-0.38, 0.07)	-0.04 (-0.27, 0.20)
	STEC	4.77 (0.46)	0.01 (-0.02, 0.52)	0.01 (-0.03, 0.45)	6.33 (1.16)	-0.01 (-0.14, 0.15)	0.05 (-0.20, 0.17)
	Shigella	4.56 (0.99)	0.06 (0.01, 0.12)	-0.01 (-0.05, 0.04)	5.77 (1.26)	-0.07 (-0.15, 0.01)	0.00 (-0.07, 0.09)
Viruses	Norovirus	5.86 (1.44)	0.05 (-0.10, 0.27)	-0.03 (-0.19, 0.17)	5.42 (1.25)	0.03 (-0.17, 0.15)	-0.04 (-0.20, 0.11)
	Sapovirus	5.63 (1.88)	0.04 (-0.09, 0.15)	-0.06 (-0.18, 0.03)	5.67 (1.50)	0.02 (-0.12, 0.27)	0.21 (0.07, 0.57)
	Adenovirus 40/41	4.90 (1.16)	0.00 (-0.13, 0.14)	0.02 (-0.11, 0.15)	5.42 (1.45)	-0.19 (-0.49, -0.02)	-0.05 (-0.29, 0.19)
	Astrovirus	5.83 (2.20)	0.03 (-0.10, 0.23)	0.01 (-0.14, 0.20)	5.19 (1.72)	-0.01 (-0.12, 0.13)	0.06 (-0.02, 0.14)
	Rotavirus	4.87 (0.94)	-0.09 (-0.23, 0.04)	-0.04 (-0.16, 0.14)	5.45 (1.84)	-0.00 (-2.49, 0.10)	0.00 (-1.11, 0.12)
Protozoa	*Giardia*	4.73 (1.56)	-0.02 (-0.12, 0.11)	-0.03 (-0.15, 0.15)	4.89 (1.70)	-0.03 (-0.11, 0.22)	0.03 (-0.21, 0.10)
	E. bieneusi	5.23 (2.47)	-0.01 (-0.05, 0.02)	0.01 (-0.03, 0.05)	5.90 (1.63)	-0.04 (-0.19, 0.10)	0.09 (-0.04, 0.32)
	Cryptosporidium	5.01 (1.98)	-0.03 (-0.08, 0.03)	-0.04 (-0.10, 0.02)	5.00 (1.36)	0.16 (0.04, 0.28)	-0.00 (-0.09, 0.13)

Table S6 continued.

		12 months (n=826)				
		Quantity*	Quantity difference† (95% CI)			
	Pathogen	Mean (SD)	WASH	IYCF	WASH + IYCF	*p* for heterogeneity
Bacteria	EAEC	6.07 (1.42)	0.09 (-0.19, 0.40)	-0.12 (-0.35, 0.24)		
	ETEC	6.17 (1.59)	0.15 (-0.20, 0.44)	0.03 (-0.30, 0.31)		
	aEPEC	5.51 (1.14)	0.32 (-0.12, 0.57)	0.16 (-0.19, 0.42)	-0.10 (-0.42, 0.29)	0.02
	Campylobacter spp.	5.36 (1.14)	-0.04 (-0.28, 0.19)	0.26 (0.08, 0.55)		
	tEPEC	6.02 (1.28)	-0.01 (-0.18, 0.19)	-0.15 (-0.37, 0.03)		
	STEC	5.56 (0.81)	0.00 (-0.15, 0.37)	-0.04 (-0.48, 0.13)		
	Shigella	5.66 (1.15)	0.03 (-0.17, 0.29)	-0.12 (-0.60, 0.19)		
Viruses	Norovirus	5.23 (1.19)	0.14 (-0.04, 0.31)	-0.02 (-0.17, 0.17)		
	Sapovirus	5.31 (1.58)	-0.03 (-0.16, 0.07)	0.11 (-0.01, 0.23)		
	Adenovirus 40/41	5.57 (1.88)	0.02 (-0.07, 0.20)	0.01 (-0.11, 0.14)		
	Astrovirus	6.34 (1.91)	0.12 (0.03, 0.22)	0.04 (-0.04, 0.14)		
	Rotavirus	4.95 (1.51)	0.01 (-0.04, 0.11)	0.00 (-0.08, 0.07)		
Protozoa	*Giardia*	6.38 (1.96)	-0.05 (-0.51, 0.34)	0.13 (-0.32, 0.49)	-0.51 (-0.85, -0.07)	0.02
	E. bieneusi	5.46 (1.45)	-0.20 (-0.47, -0.08)	-0.09 (-0.18, 0.20)		
	Cryptosporidium	5.20 (1.39)	0.01 (-0.24, 0.10)	0.15 (-0.10, 0.24)		

*Mean quantity measured in log-copy numbers per gram of stool among positive stools only

†Quantity difference includes positive and negative stools and is adjusted for age in days of sample collection

Table S7. Differences in age-specific pathogen group scores in WASH vs. non-WASH and IYCF vs. non-IYCF treatment arms among 2,181 stool samples from 992 children in the SHINE EED substudy.

Pathogen scores*	Age (months)	Mean (SD) score		Score difference† (95% CI)	Mean (SD) score		Score difference† (95% CI)
		WASH	non-WASH	WASH vs. non-WASH	IYCF	non-IYCF	IYCF vs. non-IYCF
All pathogens	1 & 3	1.0 (1.10)	1.0 (1.11)	0.02 (-0.13, 0.21)	0.97 (1.04)	1.1 (1.16)	-0.09 (-0.25, 0.10)
	6	2.6 (1.46)	2.5 (1.46)	0.02 (-0.18, 0.22)	2.6 (1.48)	2.5 (1.44)	-0.00 (-0.23, 0.16)
	12	2.9 (1.44)	3.1 (1.41)	-0.13 (-0.34, 0.06)	3.0 (1.45)	3.0 (1.40)	-0.01 (-0.18, 0.18)
Bacteria	1 & 3	0.7 (0.87)	0.6 (0.82)	0.04 (-0.08, 0.20)	0.6 (0.80)	0.7 (0.87)	-0.08 (-0.20, 0.06)
	6	2.0 (1.17)	1.9 (1.18)	0.02 (-0.14, 0.19)	2.0 (1.19)	2.0 (1.16)	-0.02 (-0.20, 0.11)
	12	2.0 (1.15)	2.0 (1.00)	0.01 (-0.14, 0.15)	2.0 (1.05)	2.0 (1.09)	-0.01 (-0.16, 0.13)
Viruses	1 & 3	0.3 (0.53)	0.3 (0.55)	-0.02 (-0.10, 0.05)	0.3 (0.55)	0.3 (0.54)	0.01 (-0.07, 0.08)
	6	0.3 (0.49)	0.3 (0.56)	-0.04 (-0.13, 0.01)	0.3 (0.55)	0.3 (0.50)	0.04 (-0.05, 0.09)
	12	0.3 (0.53)	0.3 (0.51)	0.03 (-0.03, 0.10)	0.3 (0.52)	0.3 (0.52)	0.01 (-0.05, 0.08)
Parasites	1 & 3	0.1 (0.23)	0.1 (0.24)	-0.00 (-0.02, 0.03)	0.1 (0.22)	0.1 (0.25)	-0.01 (-0.05, 0.01)
	6	0.3 (0.53)	0.3 (0.53)	0.03 (-0.02, 0.11)	0.3 (0.54)	0.3 (0.51)	0.01 (-0.06, 0.07)
	12	0.5 (0.68)	0.7 (0.75)	-0.17 (-0.27, -0.09)	0.6 (0.72)	0.7 (0.73)	-0.01 (-0.09, 0.10)

*Number of pathogens in group detected per stool sample among stools collected at 6 and 12 months of age
†Adjusted for age in days of sample collection

Table S8. Adjusted pathogen prevalence differences associated with WASH and IYCF interventions compared to control among 1,547 stool samples collected at 6 and 12 months of age from 933 children in the SHINE EED substudy.

	Pathogen	Percent stools positive at 6 months N=721 n (%)	Percent stools positive at 12 months N=826 n (%)	Prevalence difference* at 6 & 12 months (95% CI) WASH	IYCF
Bacteria	EAEC	514 (71.3)	526 (63.7)	0.01 (-0.03, 0.05)	-0.02 (-0.07, 0.02)
	ETEC	242 (33.6)	336 (40.7)	-0.01 (-0.07, 0.04)	-0.00 (-0.06, 0.05)
	aEPEC	215 (29.8)	281 (34)	0.01 (-0.04, 0.06)	0.03 (-0.02, 0.08)
	Campylobacter spp.	230 (31.9)	267 (32.3)	-0.01 (-0.07, 0.05)	0.05 (-0.00, 0.11)
	tEPEC	93 (12.9)	98 (11.9)	-0.02 (-0.06, 0.02)	-0.04 (-0.07, 0.00)
	STEC	33 (4.6)	63 (7.6)	-0.00 (-0.03, 0.02)	-0.01 (-0.03, 0.02)
	Shigella	16 (2.2)	55 (6.7)	-0.01 (-0.03, 0.01)	-0.01 (-0.03, 0.01)
Viruses	Norovirus	76 (10.5)	109 (13.2)	0.02 (-0.02, 0.06)	-0.01 (-0.05, 0.03)
	Sapovirus	52 (7.2)	60 (7.3)	0.00 (-0.03, 0.03)	0.03 (0.00, 0.05)
	Adenovirus 40/41	59 (8.2)	52 (6.3)	-0.02 (-0.05, 0.01)	0.00 (-0.02, 0.03)
	Astrovirus	13 (1.8)	21 (2.5)	0.01 (-0.00, 0.03)	0.01 (-0.00, 0.02)
	Rotavirus	12 (1.7)	17 (2.1)	-0.00 (-0.01, 0.01)	-0.00 (-0.02, 0.01)
Protozoa	*Giardia*	112 (15.5)	238 (28.8)	-0.04 (-0.09, 0.00)	-0.01 (-0.05, 0.03)
	E. bieneusi	49 (6.8)	151 (18.3)	-0.04 (-0.07, -0.00)	0.01 (-0.02, 0.04)
	Cryptosporidium	40 (5.5)	138 (16.7)	0.00 (-0.03, 0.04)	0.01 (-0.03, 0.04)

*Adjusted for age in days of sample collection, and any covariates with p<0.2 in bivariable analysis for each outcome.

Table S9. Adjusted differences in pathogen group scores per stool sample in WASH vs. non-WASH and IYCF vs. non-IYCF treatment arms among 1,547 stool samples collected at 6 and 12 months of age from 933 children in the SHINE EED substudy.

	Mean (SD) score		Score difference† (95% CI)	Mean (SD) score		Score difference† (95% CI)
Pathogen scores*	WASH	non-WASH	WASH vs. non-WASH	IYCF	non-IYCF	IYCF vs. non-IYCF
All pathogens	2.8 (1.46)	2.8 (1.46)	-0.06 (-0.21, 0.07)	2.8 (1.46)	2.8 (1.46)	-0.02 (-0.17, 0.12)
Bacteria	2.0 (1.16)	2.0 (1.09)	0.01 (-0.11, 0.12)	2.0 (1.16)	2.0 (1.09)	-0.03 (-0.16, 0.06)
Viruses	0.3 (0.51)	0.3 (0.53)	0.01 (-0.05, 0.05)	0.3 (0.51)	0.3 (0.53)	0.02 (-0.03, 0.07)
Parasites	0.4 (0.63)	0.5 (0.69)	-0.07 (-0.14, -0.02)	0.4 (0.63)	0.5 (0.69)	-0.00 (-0.06, 0.06)

*Number of pathogens in group detected per stool sample among stools collected at 6 and 12 months of age

†Adjusted for age in days of sample collection

Table S10. Secondary per-protocol analysis: pathogen prevalence differences associated with WASH and IYCF interventions compared to control among 1,384 stool samples collected at 6 and 12 months of age from 820 children who received the interventions with high fidelity† in the SHINE EED substudy.

	Pathogen	Percent stools positive at 6 months N=639	Percent stools positive at 12 months N=745	Prevalence difference* at 6 & 12 months (95% CI) WASH	IYCF
Bacteria	EAEC	455 (71.2)	478 (64.2)	0.03 (-0.02, 0.07)	-0.03 (-0.08, 0.01)
	ETEC	219 (34.3)	304 (40.8)	-0.01 (-0.07, 0.06)	-0.01 (-0.07, 0.05)
	aEPEC	192 (30.0)	245 (32.9)	0.03 (-0.02, 0.08)	0.02 (-0.03, 0.07)
	Campylobacter spp.	205 (32.1)	236 (31.7)	0.00 (-0.06, 0.06)	0.05 (-0.00, 0.11)
	tEPEC	81 (12.7)	92 (12.3)	-0.02 (-0.06, 0.02)	-0.03 (-0.07, 0.01)
	STEC	27 (4.2)	59 (7.9)	-0.01 (-0.04, 0.01)	-0.00 (-0.03, 0.02)
	Shigella	12 (1.9)	47 (6.3)	-0.00 (-0.02, 0.02)	-0.01 (-0.03, 0.01)
Viruses	Norovirus	67 (10.5)	102 (13.7)	0.02 (-0.01, 0.06)	-0.02 (-0.06, 0.02)
	Sapovirus	41 (6.4)	48 (6.4)	0.01 (-0.02, 0.03)	0.03 (0.01, 0.06)
	Adenovirus 40/41	49 (7.7)	49 (6.6)	-0.02 (-0.05, 0.01)	0.01 (-0.02, 0.04)
	Astrovirus	12 (1.9)	19 (2.6)	0.01 (-0.00, 0.02)	0.01 (-0.01, 0.02)
	Rotavirus	11 (1.7)	16 (2.2)	0.00 (-0.02, 0.02)	-0.00 (-0.02, 0.01)
Protozoa	*Giardia*	96 (15.0)	215 (28.9)	-0.05 (-0.09, 0.00)	-0.01 (-0.06, 0.04)
	E. bieneusi	41 (6.4)	140 (18.8)	-0.03 (-0.07, 0.01)	0.00 (-0.03, 0.04)
	Cryptosporidium	34 (5.3)	122 (16.4)	0.00 (-0.03, 0.04)	0.01 (-0.03, 0.04)

*Adjusted for age in days of sample collection, and any covariates with p<0.2 in bivariable analysis for each outcome.

†High fidelity defined as received all 9 intervention modules [4]

Supplemental data analysis methods: observational associations between enteropathogens and growth

To assess whether the observed enteric infections in SHINE were associated with LAZ outcomes, we used multivariable linear regression for LAZ outcomes at 12 and 18 months of age, adjusting for WASH and IYCF intervention randomization groups. Pathogen exposures were dichotomized to at least one detection vs. no detections in all 1, 3, 6, and 12 month stools. Missing stool samples or qPCR results at any of these time points were assumed to be negative for all pathogens. In a sensitivity analysis, we restricted the analysis to exposures in the 6 and 12 month stools among the subset of children with both stools analyzed by qPCR to account for confounding by age of stool sample collection and missing stool collections (Table S11). Using similar models, we further estimated the associations between 12 and 18 month LAZ outcomes and the mean number of pathogens, bacteria, viruses, and parasites detected per stool sample. Effects were estimated per additional pathogen detected on average (Table S12). All models were adjusted for birth weight, sex, maternal height, maternal mid-upper arm circumference, prematurity, wealth quintile, detection of other enteropathogens, and randomization group.

Table S11. Associations between enteropathogen detections and length-for-age z-scores at 12 and 18 months among 971 children in the SHINE EED substudy with LAZ measurements at 12 and/or 18 months.

	Among all children with at least one stool (N=971)			Among children with both 6 and 12 month stools (N=614)		
Pathogen	N (%) with 1+ detections*	12 month LAZ difference† (n=899)	18 month LAZ difference† (n=962)	N (%) with 1+ detections‡	12 month LAZ difference† (n=611)	18 month LAZ difference† (n=609)
EAEC	798 (82.2)	-0.02 (-0.19, 0.15)	-0.01 (-0.16, 0.14)	555 (90.4)	-0.09 (-0.32, 0.14)	0.01 (-0.24, 0.25)
ETEC	512 (52.7)	-0.05 (-0.19, 0.08)	-0.01 (-0.15, 0.12)	377 (61.4)	-0.10 (-0.26, 0.05)	-0.06 (-0.21, 0.09)
aEPEC	459 (47.3)	-0.07 (-0.20, 0.05)	-0.06 (-0.18, 0.07)	333 (54.2)	-0.01 (-0.16, 0.14)	-0.03 (-0.18, 0.12)
Campylobacter spp.	441 (45.4)	-0.05 (-0.17, 0.07)	-0.04 (-0.15, 0.08)	334 (54.4)	0.03 (-0.11, 0.17)	0.00 (-0.14, 0.15)
tEPEC	188 (19.4)	0.02 (-0.14, 0.19)	0.14 (-0.02, 0.29)	142 (23.1)	0.05 (-0.14, 0.23)	0.08 (-0.09, 0.25)
STEC	96 (9.9)	-0.13 (-0.32, 0.07)	-0.00 (-0.20, 0.19)	73 (11.9)	-0.15 (-0.37, 0.06)	-0.06 (-0.29, 0.17)
Shigella	75 (7.7)	-0.18 (-0.37, 0.01)	0.02 (-0.19, 0.23)	59 (9.6)	-0.25 (-0.47, -0.03)	-0.10 (-0.34, 0.13)
Norovirus	212 (21.8)	0.16 (0.02, 0.30)	-0.01 (-0.15, 0.14)	139 (22.6)	0.27 (0.10, 0.44)	0.05 (-0.14, 0.23)
Sapovirus	123 (12.7)	0.09 (-0.10, 0.28)	0.06 (-0.11, 0.23)	80 (13.0)	0.10 (-0.09, 0.29)	0.13 (-0.08, 0.33)
Adenovirus 40/41	154 (15.9)	-0.08 (-0.23, 0.07)	-0.03 (-0.19, 0.13)	89 (14.5)	-0.12 (-0.32, 0.08)	-0.09 (-0.30, 0.12)
Astrovirus	46 (4.7)	-0.27 (-0.50, -0.03)	-0.14 (-0.39, 0.11)	26 (4.2)	-0.32 (-0.55, -0.09)	-0.05 (-0.33, 0.23)
Rotavirus	96 (9.9)	0.18 (-0.06, 0.43)	0.07 (-0.13, 0.27)	21 (3.4)	0.25 (-0.28, 0.77)	0.19 (-0.30, 0.69)
Giardia	323 (33.3)	-0.01 (-0.14, 0.12)	-0.05 (-0.17, 0.07)	219 (35.7)	-0.07 (-0.23, 0.08)	-0.12 (-0.26, 0.02)
E. bieneusi	195 (20.1)	0.03 (-0.14, 0.19)	-0.02 (-0.18, 0.14)	158 (25.7)	-0.03 (-0.23, 0.18)	-0.03 (-0.22, 0.16)
Cryptosporidium	176 (18.1)	-0.15 (-0.33, 0.03)	-0.09 (-0.26, 0.08)	133 (21.7)	-0.16 (-0.37, 0.04)	-0.11 (-0.31, 0.09)

*Includes at least one detection in any stool sample collected at 1, 3, 6, and 12 months of age

†Difference in length-for-age z-score between children with at least one detection of the enteropathogen compared to children with no detections of the pathogen. Adjusted for birthweight, sex, maternal height, maternal MUAC, prematurity, wealth quintile, detection of other enteropathogens, and randomization group.

‡Includes at least one detection in any stool sample collected at 6 and 12 months of age

Table S12. Associations between mean enteropathogen scores and length-for-age z-scores at 12 and 18 months among 971 children in the SHINE EED substudy with LAZ measurements at 12 and/or 18 months.

Among all children with at least one stool (N=971)			
Pathogen scores*	Mean (SD) number of pathogens detected per stool sample	12 month LAZ difference‡ (n=899)	18 month LAZ difference‡ (n=962)
All pathogens	2.4 (1.14)	-0.04 (-0.10, 0.01)	-0.05 (-0.11, 0.01)
Bacteria	1.7 (0.87)	-0.05 (-0.13, 0.02)	-0.03 (-0.10, 0.05)
Viruses	0.3 (0.39)	0.07 (-0.08, 0.22)	-0.05 (-0.21, 0.11)
Parasites	0.4 (0.47)	-0.08 (-0.22, 0.06)	-0.11 (-0.24, 0.02)

Among children with both 6 and 12 month stools (N=614)			
Pathogen scores†	Mean (SD) number of pathogens detected per stool sample	12 month LAZ difference‡ (n=611)	18 month LAZ difference‡ (n=609)
All pathogens	2.8 (1.06)	-0.06 (-0.14, 0.01)	-0.05 (-0.12, 0.01)
Bacteria	2.0 (0.82)	-0.06 (-0.15, 0.04)	-0.02 (-0.12, 0.07)
Viruses	0.3 (0.38)	0.12 (-0.09, 0.34)	0.02 (-0.19, 0.23)
Parasites	0.5 (0.47)	-0.19 (-0.37, -0.01)	-0.18 (-0.34, -0.01)

*Number of pathogens in group detected on average in stool samples collected at 1, 3, 6, and 12 months of age

†Number of pathogens in group detected on average in stool samples collected at 6 and 12 months of age

‡Difference in length-for-age z-score per additional enteropathogen detected on average; adjusted for birthweight, sex, maternal height, maternal MUAC, prematurity, wealth quintile, detection of other enteropathogens, and randomization group.

References

1. Liu J, Gratz J, Amour C, et al. Optimization of Quantitative PCR Methods for Enteropathogen Detection. PloS One. **2016**; 11(6):e0158199.

2. Liu J, Platts-Mills JA, Juma J, et al. Use of quantitative molecular diagnostic methods to identify causes of diarrhoea in children: a reanalysis of the GEMS case-control study. Lancet Lond Engl. **2016**; 388(10051):1291–1301.

3. Liu J, Gratz J, Amour C, et al. A Laboratory-Developed TaqMan Array Card for Simultaneous Detection of 19 Enteropathogens. J Clin Microbiol. **2013**; 51(2):472–480.

4. Sanitation Hygiene Infant Nutrition Efficacy (SHINE) Trial Team, Humphrey JH, Jones AD, et al. The Sanitation Hygiene Infant Nutrition Efficacy (SHINE) Trial: Rationale, Design, and Methods. Clin Infect Dis Off Publ Infect Dis Soc Am. **2015**; 61 Suppl 7:S685-702.
